# Use of complementary and alternative medicine and self-tests by coronary heart disease patients

**DOI:** 10.1186/1472-6882-8-47

**Published:** 2008-08-04

**Authors:** Sheila Greenfield, Helen Pattison, Kate Jolly

**Affiliations:** 1 Department of Primary Care and General Practice, University of Birmingham, Edgbaston, Birmingham, B15 2TT, UK; 2 University of Aston, School of Life and Health Sciences, Aston, University, Aston Triangle, Birmingham, B4 7ET, UK; 3 Department of Public Health & Epidemiology, University of Birmingham, Edgbaston, Birmingham, B15 2TT, UK

## Abstract

**Background:**

Coronary heart disease patients have to learn to manage their condition to maximise quality of life and prevent recurrence or deterioration. They may develop their own informal methods of self-management in addition to the advice they receive as part of formal cardiac rehabilitation programmes. This study aimed to explore the use of complementary and alternative medicines and therapies (CAM), self-test kits and attitudes towards health of UK patients one year after referral to cardiac rehabilitation.

**Method:**

Questionnaire given to 463 patients attending an assessment clinic for 12 month follow up in four West Midlands hospitals.

**Results:**

91.1% completed a questionnaire. 29.1% of patients used CAM and/or self-test kits for self-management but few (8.9%) used both methods. CAM was more often used for treating other illnesses than for CHD management. Self-test kit use (77.2%,) was more common than CAM (31.7%,) with BP monitors being the most prevalent (80.0%). Patients obtained self-test kits from a wide range of sources, for the most part (89.5%) purchased entirely on their own initiative. Predictors of self-management were post revascularisation status and higher scores on 'holism', 'rejection of authority' and 'individual responsibility'. Predictors of self-test kit use were higher 'holism' and 'individual responsibility' scores.

**Conclusion:**

Patients are independently using new technologies to monitor their cardiovascular health, a role formerly carried out only by healthcare practitioners. Post-rehabilitation patients reported using CAM for self-management less frequently than they reported using self-test kits. Reports of CAM use were less frequent than in previous surveys of similar patient groups. Automatic assumptions cannot be made by clinicians about which CHD patients are most likely to self-manage. In order to increase trust and compliance it is important for doctors to encourage all CHD patients to disclose their self-management practices and to continue to address this in follow up consultations.

## Background

An on-going task for patients with coronary heart disease (CHD), as for other chronic disease sufferers, is to learn to manage their condition to maximise quality of life and prevent recurrence or deterioration [1]. Formal cardiac rehabilitation programmes are designed to introduce patients at an early stage after their cardiac event to health behaviours such as exercise, dietary modification and relaxation which they can continue to use for long term health maintenance [2]. Patients may also use other informal methods of self-management which can involve them carrying out more complex behaviours usually performed by healthcare practitioners [3]. For CHD patients these could include making decisions about medication management or monitoring blood pressure and patients may not discuss these with their doctor [4,5].

The increasing availability of complementary and alternative medicine (CAM) in the UK [6] has significant self-management implications for CHD patients. There is increasing evidence that CAM, particularly herbal medicine, can be effective in the management of cardiovascular problems [7,8]. However it has been reported that some herbal medicines can cause adverse reactions with orthodox CHD medication [9,10]. Existing studies on CAM use by CHD patients have been undertaken in healthcare contexts outside the UK, the majority being from the USA [5,11-20]. Other work has been carried out in Canada [21-23], Spain [24] and Germany [25]. These studies suggest that CHD patients use CAM for a variety of reasons e.g. for specific cardiovascular problems, to manage other medical conditions or for general health maintenance, but have found very different rates of overall CAM use ranging from 12%–85%. Some studies have identified social, psychological or medical factors as predictors of CAM use among CHD patients [5,16,17,19,23,24]. Different attitudes and behaviours around CAM in different healthcare contexts mean that in some settings it may be considered more acceptable to use CAM or to disclose use and it is therefore difficult to generalise the results of previous work to the UK setting.

A further important development for the self-management practices of CHD patients in the UK is the increasing number and availability of self-test kits for diagnosis or symptom monitoring [26-29]. These include blood pressure, cholesterol and body fat monitors that enable patients to monitor aspects of their own cardiovascular health without reference to a healthcare professional. The extent to which the UK general public or specific patient groups use these for self-management and the characteristics of users has been little explored. In a UK study in which medical students were asked about home test kit use, cholesterol, blood pressure and pregnancy tests were used by a minority, although the specific number of users was not given [30]. Results from a general public survey indicated that 32% of people had ever bought a self-test kit, including pregnancy testing, of some kind [31]. More recently Ryan et al [32] found that 15% of respondents to a survey asking about self-tests had used a test other than a pregnancy test. Studies of the prevalence of patient initiated purchase of blood pressure monitors in the general population found rates of 7.5% in the USA [33], 9% in the UK [34] and up to 17.0% in Germany [35,36]. Blood pressure monitors are commonly purchased on the individual's own initiative, rather than being recommended by a doctor [37,38]. The study described here set out to explore the use of CAM, self-test kits and attitudes towards health of a group of UK CHD patients one year after they had been referred for cardiac rehabilitation.

## Methods

The study sample consisted of patients who took part in a randomised controlled trial (RCT) of home versus hospital based cardiac rehabilitation [39]. The 525 trial participants had been referred to the cardiac rehabilitation programme in one of four hospitals in the West-Midlands Health Region, UK, in the 2 year period from 1^st ^February 2002 following a myocardial infarction, percutaneous transluminal coronary angioplasty (PTCA) or coronary artery bypass graft (CABG) within the previous 12 weeks. 475 (91.5% of live patients) were followed-up at 12 months and 463 (97.4%) were given a questionnaire exploring self-management behaviour and attitudes. Participants who did not speak and or read English were not given a questionnaire. The questionnaire was self-completed or if assistance was required it was administered by a nurse when patients attended their hospital follow-up appointment. Patients were asked to give yes/no responses to two questions about CAM use, 'Have you used any alternative or complementary therapies/medicines for your heart problems?' and 'Have you ever used any alternative/complementary therapies/medicines for any other illnesses?'. No specific definition of what CAM could include was provided, but if respondents answered yes to either of these questions they were asked in an open question to say which therapies/medicines they had used and which illnesses they had used them for. They were also asked whether they had used a blood pressure, cholesterol or body fat monitor or any other self- test kits and why and where they had purchased them.

Attitudes to CAM and health were explored based on a questionnaire developed by Siahpush [40,41] which had eight scales. Each question was scored on a five-point Likert scale, (strongly agree, agree, don't know, disagree, strongly disagree). Socio-demographic details for each patient (gender, age, ethnicity, CHD diagnosis, type of rehabilitation programme attended) were obtained from the RCT data [39]. The score for each scale was calculated as the average of the z-scores of the items composing that scale. The mean results for the raw scores are presented. Mean Z-scores for the scales of attitude to health and CAM by gender, age group and ethnicity were compared using a non-paired t-test. The results of the scales and demographic characteristics were entered into a binary logistic regression model to estimate the effect of these factors on the use of CAM, self-testing and self-management strategy use.

Responses to an open question which asked patients why they had used a self-test were analysed by selecting and reorganising responses according to themes [42]. Ethics approval for the study was obtained from the local research ethics committees.

## Results

### Patient characteristics

422 patients (422/463; 91.1%) completed a questionnaire. Table 1 shows that the majority were male, of white ethnic origin, aged under 65 and with a diagnosis of MI or PTCA. Similar numbers of patients were referred to home or hospital cardiac rehabilitation programmes.

**Table 1:** Patients' socio-demographic characteristics

**Characteristic**	**No. (%)**
**Gender**	
Male	322 (76.3)
Female	100 (23.7)
**Ethnicity**	
White	349 (82.7)
Non white	73 (17.3)
**Age: Range 29 to 88 Mean = 61.12 (SD 10.85)**	
< = 54	114 (27.0)
55–64	135 (32.0)
65–74	118 (28.0)
75+	55 (13.0)
**Diagnosis group**	
MI	202 (47.9)
PTCA	170 (40.3)
CABG	50 (11.8)
**Rehabilitation type**	
Home	208 (49.3)
Hospital	214 (50.7)

### Self-management

123 patients (123/422, 29.1%) used some form of the two types of self-management investigated. Of these 39 (31.7%, 39/123) said they used CAM, 95 (77.2%, 95/123) used a self-test, 11, (8.9%, 11/123) had used both. Overall, self-test use was more common than CAM use, with 22.5% (95/422) of all the study patients having used one compared with 9.2% (39/422) of patients using CAM. Only one third of CAM users (28.2%, 11/39) said they used it to manage their cardiac problems, whereas 82.1% (32/39) used it for treating other illnesses. Patients were asked to state which therapies they used and two were mentioned for the management of their heart condition: vitamins and dietary supplements (6 patients), exercises (2 patients), not specified (3 patients). For the management of other conditions, patients often mentioned only the condition that they used the CAM for, but six CAMS were specified: acupuncture (8 patients), homeopathy (6 patients), chiropractic (1 patient), dietary supplements (1 patient), massage and rubbing for aches and pains (3 patients), exercises (1 patient).

Patients were asked if they had used a blood pressure monitor, a cholesterol monitor, or a body fat monitor and to specify if they had used any other self-test kits. Of the 95 patients who used a self-test, 79 (79/95, 83.1%) had monitors for blood pressure, 22 (22/95, 32.1%) for blood sugar, 6 (6/95, 6.3%) for cholesterol, 2 (2/95, 2.1%) for body fat and 1 (1/95, 1.0%) for heart rate. Eighty-nine patients described where they had obtained them. Although most commonly this was from a pharmacy (47.2%, 42/89), other sources were mail order (13.5%, 12/89), family and friends (12.4%, 11/89), large high street retailers (3.4%, 3/89), the internet (2.2%, 2/89) and the workplace (1.1%, 1/89). Fifteen patients said the self-test had been provided either by their GP or the hospital, but for the vast majority of patients (89.5%, 86/89) use was entirely self-initiated. An open question asked patients why they had used a self-test. Reasons given could be grouped into 6 main themes; to regularly monitor specific aspects of their health (mentioned by 56 patients), recommendation by their GP or hospital (9 patients), to detect changes as a result of exercise, diet or feeling unwell (4 patients), recommendation by a family member (4 patients), directly following their cardiac event (3 patients) or to avoid having to keep going to their GP (1 patient).

### Attitudes to health and CAM

Table 2 shows that for all of the scales, except individual responsibility and consumerism, the mean score was greater in the group of self-management users, the difference being statistically significant for four of the eight scales. This suggests that patients who used self-management were significantly more likely to be dissatisfied with the way doctors behave towards patients (scale 3); to take a 'holistic' attitude towards their health (scale 5); to be less accepting of medical power and to favour equality in the doctor/patient relationship (scale 6). Mean scores for the fourth scale which showed statistically significant data (scale 7) were in the opposite direction and showed that self-management users were less likely to feel that being healthy involves effort by the individual.

**Table 2:** Comparison of attitudes to health and CAM scores of users and non-users of self-management

**Scale**	**Self-management users ** **N = 123**		**Non-users of Self-management ** **N = 299**		**p value**
			
	**Mean**	**SD**	**Mean**	**SD**	
**1. Attitude towards alternative medicine (max score 25):**					
-I think most alternative therapies do not work.	15.08	2.94	14.78	2.40	0.3
-I would recommend alternative medicines to anyone of my friends who might get ill					
-I would never use the services of alternative therapists.					
-I trust most alternative therapists.					
-I think most alternative therapists are quacks					

**2. Dissatisfaction with medical outcomes (max score 30):**					
-I feel confident that doctors are able to cure most illnesses.	13.44	3.78	12.85	3.36	0.1
-Doctors can rarely do much for sick people.					
-Many times doctors don't help their patients get well.					
-I have very little confidence in the ability of most GPs to give the correct diagnosis.					
-Doctors often over-prescribe drugs					
-I think conventional medicine is unable to treat a lot of illnesses.					

**3. Dissatisfaction with medical encounter *(max score 35):***					
-Most doctors pay a lot of attention to the individual needs of their patients.	18.75	5.04	17.57	4.45	0.03
-Most doctors have a lot of respect for their patients.					
-Most doctors listen carefully to their patients during consultations.					
-Most doctors do not give enough explanations to their patients.					
-Most doctors spend too little time with their patients.					
-Doctors have too much power over their patients.					
-Most doctors are too authoritative in their relationship with their patients					

**4. Natural remedies (max score 30):**					
-I prefer natural remedies to chemical drugs	15.85	2.64	15.66	2.56	0.5
-Most prescription drugs have negative side effects.					
-Additives such as preservatives and artificial colouring are harmful to health.					
-Most prescription drugs obtained from pharmacies are effective.					
-I think it is necessary for people who are ill to take medicines that doctors prescribe.					
-Without medications that doctors prescribe, illnesses can never be cured.					

**5. Holism (max score 20):**					
-Health is more than just keeping your body fit	15.09	1.96	14.55	1.70	0.009
-Health is about harmonising your body, mind and spirit.					
-Imbalances in a person's life are the major causes of illnesses					
-Treatments should concentrate only on symptoms rather than the whole person.					

**6. Rejection of authority (max score 25):**					
-Health practitioners should treat their patients as equals	18.53	2.32	17.80	2.01	0.003
-Patients should be able to have an input in what remedies health practitioners prescribe.					
-Patients should have some say over what goes on during consultations.					
-Health practitioners should act as authority figures in their relationship with patients.					

**7. Individual responsibility (max score 15):**					
-Achieving good health requires a change of lifestyle.	11.28	1.93	11.69	1.67	0.03
-Achieving good health requires hard work and commitment					
-We are what we are because of the choices we make.					

**8. Consumerism (max score 5):**					
-It's good that nowadays we have so many different types of therapies to choose from.	3.84	0.70	3.88	0.73	0.6

Women had a higher 'consumerism' score than men indicating a positive attitude to choice in healthcare (p = 0.01) (Table 3). Non-white patients were more dissatisfied than white patients with 'medical outcomes' i.e. the efficacy of doctors and conventional medicine (p < 0.001), the 'medical encounter' i.e. doctors' behaviour and attitudes to patients within the consultation (p = 0.001) and were more positive towards natural remedies' (p = 0.02). Patients aged under 65 were more 'dissatisfied with the 'medical encounter' than those 65 and over (p = 0.002).

**Table 3:** Patients' socio-demographic characteristics by attitudes to health

	**Alternative medicine**	**Medical outcomes**	**Medical encounters**	**Natural remedies**	**Holism**	**Rejection of authority**	**Responsibility**	**Consumerism**
**Gender**								
Males mean (SD)	-0.02 (1.01)	-0.01 (0.96)	0.03 (0.98)	-0.009 (1.0)	0.002 (1.02)	-0.05 (1.04)	-0.04 (0.98)	-0.06 (1.02)
Females mean (SD)	0.08 (0.98)	0.04 (1.12)	-0.11 (1.07)	-0.03 (1.03)	-0.005 (0.93)	0.17 (0.84)	0.13 (1.05)	0.22 (0.88)
Mean Difference	-0.10	-0.049	0.14	-0.038	0.007	-0.22	-0.17	-0.28*
95% CI of mean diff	-0.34, 0.14	-0.29, 0.19	-0.10, 0.38	-0.27, 0.20	-0.23, 0.24	-0.45, 0.02	-0.40, 0.07	-0.49, -0.07
p-value	0.4	0.7	0.2	0.8	1.0	0.07	0.2	0.02

**Ethnicity**								
Whites mean (SD)	0.04 (0.95)	-0.09 (0.97)	-0.07 (0.99)	-0.05 (0.99)	-0.001 (0.98)	0.017 (1.00)	-0.018 (1.05)	0.008 (1.00)
Non-whites mean (SD)	-0.22 (1.22)	0.48 (1.02)	0.35 (0.99)	0.26 (1.07)	0.007 (1.10)	-0.08 (1.00)	0.09 (0.68)	-0.04 (1.01)
Mean Difference	0.27	-0.57*	-0.43*	-0.31*	-0.008	0.10	-0.11	0.05
95% CI of mean diff	-0.06, 0.59	-0.84, -0.31	-0.69, -0.17	-0.58, -0.05	-0.27, 0.25	-0.16, 0.36	-0.31, 0.09	-0.22, 0.31
p-value	0.05	<0.001	0.001	0.02	1.0	0.5	0.4	0.7

**Age group**								
<65 yrs mean (SD)	0.07 (1.01)	0.03 (1.04)	0.13 (1.02)	0.02 (1.04)	0.01 (1.03)	-0.02 (1.08)	-0.05 (1.01)	-0.013 (1.03)
65+ yrs mean (SD)	-0.11 (0.98)	-0.04 (0.92)	-0.20 (0.93)	-0.03 (0.94)	-0.02 (0.95)	0.02 (0.87)	0.07 (0.98)	0.02 (0.97)
Mean Difference	0.19	0.06	0.33*	0.05	0.03	-0.04	-0.12	-0.03
95% CI of mean diff	-0.02, 0.39	-0.14, 0.27	0.13, 0.53	-0.15, 0.25	-0.16, 0.23	-0.24, 0.16	-0.32, 0.08	-0.17, 0.23
p-value	0.07	0.5	0.001	0.6	0.7	0.7	0.2	0.8

* p < 0.05.

Attitudes to health presented as z-scores.

### Factors influencing use of self-management

Multiple logistic regression was carried out to measure the effect of age, gender, ethnicity, diagnosis and health attitudes on the use of self-management and on CAM and self-test use separately, and overall self-management (CAM or self-test use). Age, gender and ethnicity did not predict use of self-management strategies, however patients post revascularisation were significantly more likely to use a self-management strategy (compared with patients post-heart attack) OR 1.95 (95% CI 1.18, 3.23) (Table 4). Holism, rejection of authority and individual responsibility scores were significantly associated with self-management use. A similar picture emerged for use of self-tests, although rejection of authority score was not significant (Table 4). When demographic characteristics and attitudes to alternative medicine score (scale 1) were entered into a regression model, the only predictor of CAM use was attitude to alternative medicine OR 1.7 (95% CI 1.15, 2.5). When the other scores for attitudes to alternative medicine and health (scales 2–8) were added to the model, no characteristics predicted use of CAM.

**Table 4:** Multiple regression analysis exploring the effect of age, gender, ethnicity, diagnosis and attitudes to health scales on use of self-management and self-test kits

	**Self-management users ** (CAM & self-test kit users, N = 123)			**Self-test kits ** (Self-test kit users N = 95)		
		
	**OR**	**95% CI**	**p value**	**OR**	**95% CI**	**p value**
Age	1.0	0.98, 1.02	0.7	0.99	0.96, 1.01	0.3
Females^1^	0.74	0.37, 1.48	0.4	0.88	0.42, 1.83	0.7
Ethnic non-whites^2^	1.58	0.80, 3.12	0.2	1.33	0.65, 2.75	0.6
Post -revascularisation^3^	1.95	1.18, 3.23	0.009	2.04	1.19, 3.49	0.01
Attitude towards alternative medicine	1.13	0.87, 1.47	0.4	1.07	0.82, 1.40	0.6
Dissatisfaction with medical outcomes	1.05	0.76, 1.44	0.8	1.02	0.73, 1.42	0.9
Dissatisfaction with the medical encounter	1.25	0.92, 1.69	0.2	1.18	0.86, 1.63	0.3
Natural remedies	0.92	0.70, 1.20	0.5	0.82	0.61, 1.09	0.2
Holism	1.41	1.08, 1.84	0.01	1.44	1.09, 1.91	0.01
Rejection of authority	1.30	1.0, 1.69	0.05	1.21	0.92, 1.58	0.2
Individual responsibility	0.69	0.53, 0.90	0.006	0.69	0.53, 0.91	0.009
Consumerism	1.0	0.78, 1.29	1.0	0.43	0.71, 1.19	0.5

^1 ^base case males; ^2 ^base case whites; ^3 ^base case post-MI

## Discussion

This study has shown that one year after a cardiac event just over a quarter of patients (29.4%) were using either CAM and/or a self-test for self-management of their health. This is similar to rates of CAM or over-the-counter medication use among the general UK population [43,44]. CAM use among CHD patients has not been previously explored in the UK context and CAM use was low (8.1%) compared with studies in other countries, and 6 therapies, acupuncture, homeopathy, vitamins and dietary supplements, chiropractic, exercises and massage were specifically mentioned by respondents. A number of factors may help to account for this. In some of these studies patients were specifically asked about use of particular CAM therapies [14,16,19], sometimes the definition of CAM was very broad including for example vitamins, prayer or spiritual healing [14,19,21]. In the current study patients were not given a definition of CAM or a prepared list of CAM therapies/medicines but were simply asked whether they had used it and to list any therapies/medicines used. Definitions of CAM can vary widely and this may have led to an underestimation of CAM use as a whole. For example it may be that respondents did not automatically think of particular behaviours or practices as CAM and therefore did not mention them. Providing respondents with a checklist of therapies/medicines might have produced a higher rate of CAM use. The questionnaire was frequently administered by a nurse in a hospital setting, and some patients might therefore have been reluctant to disclose CAM use [5]. The patients were given the questionnaire one year after their cardiac event, i.e. at a comparatively early stage and it may be that higher rates of CAM use would be seen subsequently as patients further develop their self-management strategies [45].

In addition it has been suggested that in the UK context CAM use may vary among different ethnic groups [46]. The socio-demographic characteristics of patients recruited to the original trial [39], where patients who did not speak either English or Punjabi (the predominant minority language in the locality), were excluded and for the current study in which patients who did not speak or read English were not given a questionnaire, may have influenced eventual estimates of CAM use. However, only 25 participants of the original trial described their ability to read English as 'not at all' or 'slightly', so their exclusion is unlikely to have caused much bias, although some selection bias will have occurred as 105 people from ethnic minority groups were not eligible for the trial due to language restrictions. The general finding that CAM was more commonly used for general, rather than cardiovascular health management is similar to the results of recent USA studies [5,19]. It is important however that patients are aware that the type of CAM therapy they use for other conditions might impact on the management of their cardiovascular problem [9,10].

This is the first UK study to focus on the use of self-test kits for self-management by CHD patients and the characteristics of users. In the current study self-test use was more common than CAM, amounting to 22.7% of all study patients. For the vast majority, the impetus was entirely their own and not sanctioned by a healthcare professional. This reflects how patients are using new technologies to take on responsibility for their own health. It is not surprising that blood pressure and blood sugar monitors were the type of self-test most commonly used by this group of patients as they are easily accessible [29], relevant for their condition and are measurements they are accustomed to having regularly checked by health care staff. Home monitoring of blood pressure under medical guidance and control has been suggested as a method of increasing patient compliance and individual management of health [47]. However, where the initiative has come from patients themselves, use of a home BP monitor enables patients to monitor their own progress, compare their readings with those taken in the clinic and potentially challenge them, and identify and report any between clinic visit changes to their doctor. This has the potential to cause a shift in the health care professional/patient power relationship [26].

Previously identified socio-demographic predictors in a general population of being more likely to self-manage are high educational level and extent of and familiarisation with medical knowledge [48]. Among the CHD patients in the current study, where the two types of self-management investigated were CAM or self-test use, the only predictor for self-management in general or self-test use was type of cardiac event. No statistically significant predictor of CAM use was identified. Patients who had a revascularisation were more likely to self-manage than those who had a heart attack, which could be explained by the two patient groups having different beliefs about the nature of their disease. Patients with angina have described the incurable nature of their disease [49], whereas patients who have had a heart attack may have understood from health care professionals that complete recovery is possible [50]. Alternatively, patients who have had a heart attack may perceive their condition to be more serious than those who have had a revascularisation and hence feel it more appropriate to rely on medical management. However three attitudinal predictors were identified; preference for a highly 'mutualistic' relationship between patient and doctor [51], a holistic approach to health and a belief that individual behaviour affects health. Although the latter two attitudes were predictors of self-test kit use, preference for mutualism was not. This suggests that whilst CHD patients may be keen to monitor their condition, they are well aware of the importance of formal healthcare.

## Conclusion

This research has gained insight into the use of CAM and self-test kits for self-management by CHD patients in the UK setting and has demonstrated that just over a quarter of patients self-managed in one or both of these ways. Patients tended to use either CAM or self-test kits rather than both methods, self-test kit use being more popular. CAM in contrast to self-testing was less likely to be used for CHD management. The study findings demonstrate how patients are independently making use of new technologies to gain information about aspects of their cardiovascular health, which could only previously have been gained from their healthcare practitioner. Although type of cardiac event and individual attitudes to health were predictors of self-management use, no socio-demographic predictors could be identified in this sample. This suggests that automatic assumptions cannot be made by clinicians about which CHD patients are most likely to self-manage.

It is important for doctors to encourage all CHD patients to disclose their self-management practices and to continue to address this in follow up consultations. It can help to identify any potential problems of adverse reactions with orthodox medication. Harnessing the patient's experience can lead to greater patient empowerment. If the technology is there, patients will use it and ignoring or disregarding the patient's experience can reduce trust and compliance.

## Competing interests

The authors declare that they have no competing interests.

## Authors' contributions

SG and KJ conceived the study and analysed and interpreted the data. HP and SG conceived the idea on which the questions relating to self-testing are based. SG wrote the first draft of the manuscript and all authors contributed to subsequent drafts and approved the final manuscript.

## Pre-publication history

The pre-publication history for this paper can be accessed here:


